# Differences in viral load among human respiratory syncytial virus genotypes in hospitalized children with severe acute respiratory infections in the Philippines

**DOI:** 10.1186/s12985-016-0565-8

**Published:** 2016-06-27

**Authors:** Francois Marie Ngako Kadji, Michiko Okamoto, Yuki Furuse, Raita Tamaki, Akira Suzuki, Irene Lirio, Clyde Dapat, Rungnapa Malasao, Mariko Saito, Gay Anne Granada Pedrera-Rico, Veronica Tallo, Socorro Lupisan, Mayuko Saito, Hitoshi Oshitani

**Affiliations:** Tohoku University Graduate School of Medicine, Sendai, Japan; Tohoku-RITM Collaborating Research Center on Emerging and Reemerging Diseases, Muntinlupa City, the Philippines; Biliran Provincial Hospital, Naval City, the Philippines; Research Institute for Tropical Medicine, Muntinlupa City, the Philippines

**Keywords:** Respiratory syncytial virus, Viral load, Genotype, Children, Pneumonia

## Abstract

**Background:**

Human respiratory syncytial virus (HRSV) is a leading viral etiologic agent of pediatric lower respiratory infections, including bronchiolitis and pneumonia. Two antigenic subgroups, HRSV-A and B, each contain several genotypes. While viral load may vary among HRSV genotypes and affect the clinical course of disease, data are scarce regarding the actual differences among genotypes. Therefore, this study estimated and compared viral load among NA1 and ON1 genotypes of HRSV-A and BA9 of HRSV-B. ON1 is a newly emerged genotype with a 72-nucleotide duplication in the G gene as observed previously with BA genotypes in HRSV-B.

**Findings:**

Children <5 years of age with an initial diagnosis of severe or very severe pneumonia at a hospital in the Philippines from September 2012 to December 2013 were enrolled. HRSV genotypes were determined and the viral load measured from nasopharyngeal swabs (NPS). The viral load of HRSV genotype NA1 were significantly higher than those of ON1 and BA9. Regression analysis showed that both genotype NA1 and younger age were significantly associated with high HRSV viral load.

**Conclusions:**

The viral load of NA1 was higher than that of ON1 and BA9 in NPS samples. HRSV genotypes may be associated with HRSV viral load. The reasons and clinical impacts of these differences in viral load among HRSV genotypes require further evaluation.

## Findings

Human respiratory syncytial virus (HRSV) is a major etiologic agent of lower respiratory tract infections, including bronchiolitis and pneumonia, and a leading cause of hospitalizations among infants in both developing and developed countries [1]. Two subgroups, HRSV-A and HRSV-B, have been described. A total of 11 HRSV-A genotypes have been identified, including GA1 to GA7 [2], SAA1 [3], NA1 and NA2 [4], and, most recently, ON1, which has a 72-nucleotide duplication in the G gene [5]. A total of 19 HRSV-B genotypes have been reported, including GB1 to GB4 [2], SAB1 to SAB3 [3], and BA, with a 60-nucleotide duplication in the G gene, which is subdivided into BA1 to BA12 [6–9]. The newly-emerged BA genotypes have replaced the previous genotypes [10]; similarly, ON1, derived from NA1 of HRSV-A, has been recently reported in many regions worldwide [11–13]. Both BA and ON1 have nucleotide duplications in the hypervariable region of the G gene. However, the effect of these duplications on the spread of these genotypes remains unknown. Although, viral load is an important factor in HRSV disease and severity [14], to date, there are no data regarding differences in HRSV viral load among different genotypes. We therefore investigated HRSV viral load among three genotypes identified in hospitalized pediatric patients.

A case series study was conducted from September 2012 to August 2013 in the Biliran Provincial Hospital, a referral hospital on Biliran Island [15] in the Philippines. Children aged <5 years admitted with an initial diagnosis of severe or very severe pneumonia based on Integrated Management of Childhood Illness criteria (IMCI) [16] were asked participation into the study and written parental consent was obtained for each participant. Severe pneumonia were defined as cases with cough and/or difficulty of breathing accompanied by chest indrawing and/or one or more danger signs including inability to drink, convulsion or lethargic/asleep. Local medical staffs were trained with a standardized protocol for nasopharyngeal swab (NPS) samples through the study period. Samples were taken using EX-swab 002 (DENKA SEIKEN Tokyo, Japan), stirred in 3 ml viral transport medium, which consists of Hanks BSS (Balanced salt solution), 0.25 % gelatin, 0.035 % sodium bicarbonate, Streptomycin (500 μg/mL) and Penicillin (500 U/mL) and Amphotericin B (250 μg/mL) and kept at 0–4 °C. Viral RNA was extracted using the QIAamp MinElute Virus Spin Kit (QIAGEN, Hilden, Germany) and cDNA was synthesized using Moloney murine leukemia virus reverse transcriptase and random primers (Thermo Scientific, Boston, MA, USA).

The first real-time polymerase chain reaction (PCR) for screening of HRSV was performed using an Applied Biosystems StepOnePlus system (Applied Biosystems, Foster City, CA, USA) as previously described, with some modifications [17] in Research Institute for Tropical Medicine (RITM), Metro Manila. The region of the N gene targeted by the real-time PCR is highly conserved among subgroups and genotypes. The detection limit of the assay was 100 copies for all genotypes.

The positive cDNA samples were shipped from RITM to laboratory at Tohoku University and viral load was defined as viral RNA copy number estimated from mean threshold cycle (Ct) values in duplicated samples obtained from the second real-time PCR with synthesized viral genomic RNA standard samples for each genotype (N gene, 658 nucleotides). For subgrouping and genotyping, the C-terminus region of the G gene was amplified and sequenced as previously described [18].

Patient characteristics, clinical symptoms, elapsed time between illness onset and specimen collection, viral coinfection (influenza virus, human rhinovirus/enterovirus, human metapneumovirus, and human parainfluenza virus) and viral load (Log_10_ RNA copies/μL) were compared among HRSV genotypes using chi-square and Kruskal-Wallis tests and one-way analysis of variance (ANOVA). The beta coefficients for associations between viral load and genotypes or other factors were adjusted by generalized linear regression. Statistical analysis was performed using IBM SPSS Statistics for Windows, version 20 (IBM Corp, Armonk, NY, USA) and a *P* value of < 0.05 was considered statistically significant.

A total of 440 clinical samples were collected. Of these, 179 (40.7 %) were positive for HRSV by the first run of the real-time PCR. Viral load of the 33 samples were not included in the analysis due to not having enough volume for the replication (*n* = 29) or negative results in the second run (*n* = 4), therefore, duplicated Ct values were obtained from 146 (81.5 %) of HRSV positive samples. Moreover, six samples were excluded due to discordant results in two replicates (*n* = 4) and failure in subgrouping (*n* = 2). A total of 140 samples were genotyped and the numbers of NA1, ON1, and BA9 genotypes were 17 (12.1 %), 51 (36.4 %), and 71 (50.7 %), respectively. One sample (0.7 %) identified as genotype GB2 was excluded from further analysis. Genotype ON1 was first detected in December in the study population. Background characteristics and symptoms of the 139 HRSV positive patients included in the analyses are shown in Table 1. Their median age was 9 months old (interquartile range, 3–17 months) and 60.4 % were male. The mean interval between illness onset and specimen collection was 4 days. No congenital heart disease or chronic lung disease were reported.

**Table 1:** Characteristics of 139 hospitalized patients due to severe acute respiratory infection by HRSV genotypes

HRSV genotype	NA1	ON1	BA9	*P* value
	*n* = 17	*n* = 51	*n* = 71	
Age in months, median [IQR]	10 [3–19]	6 [3–11]	11 [5–19]	0.026*
Males (%)	10 (58.8)	33 (64.7)	41 (57.7)	0.733**
Days between illness onset and specimen collection, median [IQR]	4 [3–5]	5 [4–7]	4 [3–7]	0.074*
Symptoms (%)				
Fever	13 (76.5)	39 (76.5)	61 (85.9)	0.361**
Cough	17 (100.0)	51 (100.0)	71 (100.0)	-
Chest indrawing	17 (100.0)	51 (100.0)	71 (100.0)	-
Difficulty of breathing	12 (70.6)	41 (80.4)	50 (70.4)	0.436**
Inability of drink	0 (0.0)	1 (2.0)	5 (7.0)	0.255**
Neurological signs	0 (0.0)	5 (9.8)	5 (7.4)	0.398**
Peripheral oxygen saturation < 90 % (%)	1 (5.9)	6 (11.8)	11 (15.5)	0.542**
Viral coinfection (%)	0 (0.0)	7 (13.7)	4 (8.4)	0.114**
Viral load (Log_10_ RNA copies/μL), median [IQR]				
All ages	6.5 [5.8–6.8]	5.4 [4.6–6.2]	5.4 [4.8–6.1]	<0.001***
< 6 months	(*n* = 6)	(*n* = 27)	(*n* = 21)	
	6.6 [6.2–7.2]	5.7 [4.7–6.4]	5.8 [5.2–6.2]	0.032*

HRSV: Human Respiratory Syncytial Virus, Neurological sign: history of convulsion or lethargic/asleep

*Kruskal-Wallis test, **chi-square test, ***one-way ANOVA

IQR: 25^th^ and 75^th^ Interquartile range, One patient positive for genotype GB2 was excluded from this analysis

Viral loads were higher in patients with genotype NA1 than in those with genotypes ON1 and BA9 (*P* = 0.0002, Table 1). Multiple regression analysis showed that younger age at admission and genotype NA1 were associated with higher HRSV viral load (Table 2). Because the NA1 viral load was also higher in infants less than 6 months of age (*P* = 0.032, Table 1), with possibly their first HRSV infections, the immunological effects of prior infection in this age group was minimal. Since genotype NA1 had been a major genotype [18] and ON1 newly introduced in this area during the study period, the effect of maternal immunity does not explain the low viral load of ON1. Therefore, the difference of viral loads among genotypes was not likely due to differences in preexisting immunity at enrollment. In addition, previous studies have shown that neutralizing antibody titer at disease onset is not associated with HRSV viral load [19]. HRSV viral load showed a strong positive correlation with younger age at admission. Young infants undergo significant anatomic and immunological changes during their development, which may affect HRSV replication patterns in different pediatric age groups.

**Table 2:** Distribution and adjusted coefficients of viral and patient factors for HRSV viral load

Variables		n	*β* (95 % CI)	*P* value
Age (months)	< 6	54	Ref.	-
	6–11.9	32	−0.21 (−0.57, 0.15)	0.264
	≥ 12	53	−0.55 (−0.92, −0.18)	0.004
Sex	Female	55	Ref.	-
	Male	84	0.28 (−0.02, 0.58)	0.072
Days between illness onset and specimen collection	< 4	45	Ref.	-
	≥ 4	94	0.15 (−0.16, 0.46)	0.347
Genotype	NA1	17	Ref.	-
	ON1	51	−1.13 (−1.53, −0.73)	< 0.001
	BA9	71	−0.92 (−1.27, −0.56)	< 0.001
Viral coinfection	No	128	Ref.	-
	Yes	11	−0.19 (−0.82, 0.44)	0.552

*HRSV* human respiratory syncytial virus, *β*: Coefficients for viral load (Log_10_ RNA copies/μL) were adjusted for the variables each other by generalized linear model. One patient positive for genotype GB2 was excluded from this analysis

Genetic differences among genotypes may explain the differences in viral load. ON1 has a 72-nucleotide insertion in the G gene [5]. This insertion is absent in the NA1 genotype and is the largest insertion described to date in this genus, which may affect the viral structure and pathogenesis. BA9, also characterized by an insertion in the G gene, also showed significantly lower viral load compared to NA1. The G protein is one of the major HRSV protective antigens and has significant roles in cell attachment [20], cell fusion [21], and immune response [22]. Insertions and duplications in the G gene may have affected the efficiency of HRSV replication in the newly emerged ON1 and BA genotypes. Detailed molecular analysis of the G protein gene sequence of several HRSV strains commonly reveal multiple short sequence repeats. The earlier report in HRSV-B of the 60-nucleotide duplication (the BA genotypes) had shown an exceptional example of repeated sequence in the G protein, which emerged and replaced previous genotypes within HRSV-B [10]. Further studies are needed to better understand why viral load differs among genotypes as well as the impact of new insertions in the G gene on HRSV pathogenesis.

Limitation of our observation is the small number of patients in NA1 group and NPS sampling by different study personnel, which may have had inter- sampling variation. Also viral load in NPS may not enhance the severity of lower respiratory infection. Others factors that may influence HRSV viral load such as, prematurity, breast feeding status and use of steroids or other immunosuppressive medication were not assessed in this study.

In the present study, age and viral genotypes were host and viral factors that independently predicted HRSV viral load. Age is a well-known predictor of HRSV disease severity [1] and is also associated with HRSV viral load. Although other studies using molecular techniques did not observe differences in viral load between HRSV subgroups [23, 24], differences in sample size, sample collection, or circulating genotypes during their study periods could explain this difference in findings. Our findings suggest the critical need for further studies to investigate potential differences in viral load among genotypes and their association with disease severity.

## Abbreviations

Ct, cycle threshold; HRSV, human respiratory syncytial virus; NPS, nasopharyngeal swab; PCR, polymerase chain reaction

## Figures and Tables

**Fig. 1:**
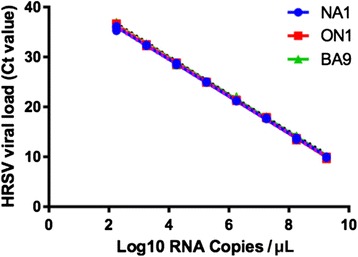
Quantitative PCR amplification of partial sequence (658 nucleotides) N genes of HRSV NA1, ON1 and BA9 genotypes. A dilution series of high-quality RNA copies was used to generate standard curves as describe in the methods. Reactions were performed in triplicate. HRSV genotypes Ct values stand for the cycle at which reporter fluorescence crosses the software-defined threshold. The linear range of threshold cycle vs RNA copies was (2 - 9) Log10 copies/μl. Solid line, linear regression of RNA copies vs Ct values. Dashed lines represent the 95 % confidence intervals for potential regression lines. The slopes and y-intercepts are -3.70, 44.35 for NA1, -3.77, 44.83 for ON1 and -3.74, 44.91 for BA9
